# Immune response to *Leishmania* antigens in an AIDS patient with mucocutaneous leishmaniasis as a manifestation of immune reconstitution inflammatory syndrome (IRIS): a case report

**DOI:** 10.1186/s12879-015-0774-6

**Published:** 2015-02-03

**Authors:** Luana Gois, Roberto Badaró, Robert Schooley, Maria Fernanda Rios Grassi

**Affiliations:** Centro de Pesquisas Gonçalo Moniz, Fundação Oswaldo Cruz (FIOCRUZ), Salvador, Bahia Brazil; Hospital Professor Edgard Santos, Universidade Federal da Bahia, Salvador, Bahia Brazil; Department of Medicine, University of California, San Diego, USA

**Keywords:** HIV, *Leishmania*, IRIS, Cytokines, Immune response

## Abstract

**Background:**

After the onset of HAART, some HIV-infected individuals under treatment present a exacerbated inflammation in response to a latent or a previously treated opportunistic pathogen termed immune reconstitution inflammatory syndrome (IRIS). Few reports of tegumentary leishmaniasis have been described in association with IRIS. Moreover, the immunopathogenesis of IRIS in association with *Leishmania* is unclear.

**Case presentation:**

The present study reports on a 29-year-old HIV-infected individual who developed mucocutaneous leishmaniasis associated with immune reconstitution inflammatory syndrome (IRIS) five months following highly active antiretroviral therapy (HAART). Severe lesions resulted in the partial destruction of the nasal septum, with improvement observed 15 days after treatment with Amphotericin B and corticosteroids. The immune response of this patient was evaluated before and after the lesions healed. IRIS was diagnosed in association with high levels of TNF-α and IL-6. Decreased production of IFN-γ and a low IFN-γ/IL-10 ratio were also observed in response to *Leishmania* antigens. After receiving anti-leishmanial treatment, the individual’s specific Th1 immune response was restored.

**Conclusion:**

The results suggest that the production of inflammatory cytokines by unstimulated T-lymphocytes could contribute to occurrence of leishmaniasis associated with IRIS.

## Background

Highly active antiretroviral therapy (HAART) has significantly benefited the majority of HIV-infected individuals. HAART results in a decrease in the plasma HIV-viral load and a partial recovery of CD4 + T-lymphocytes [1]. As a consequence, a sharp decrease is observed in morbidity and mortality rates [2]. However, some individuals under treatment experience clinical deterioration as a result of unregulated and rapid restoration of the immune response; i.e., the immune reconstitution inflammatory syndrome (IRIS). In these cases, an exacerbated inflammatory immune response against subclinical pathogens or residual antigens is observed [3].

Most cases of IRIS are associated with the *Mycobacterium avium* complex, *Mycobacterium tuberculosis*, cytomegalovirus or herpes zoster [4,5]. Few cases of tegumentary leishmaniasis as a manifestation of IRIS in patients with AIDS have been reported to date [6-8]. Furthermore, the underlying immunological mechanisms of IRIS in association with this co-infection remain unclear. The present report describes a case of severe mucocutaneous leishmaniasis as a manifestation of IRIS in an HIV-infected patient from Brazil, and evaluates his cellular immune responses to *Leishmania* antigens.

## Materials and methods

A case of mucocutaneous leishmaniasis in association with IRIS in an HIV-infected individual was recorded in 2009 at the Professor Edgar Santos University Hospital (HUPES), located in Salvador, Bahia–Brazil. The HUPES Institutional Research Review Board approved the present case report and informed written consent was obtained from the patient. Blood samples for immunological assessments were collected prior to and immediately after (the following day) the course of Amphotericin B and corticosteroid treatment. Peripheral blood mononuclear cells (PBMCs) were isolated by passage over a Ficoll-Hypaque gradient (Amersham Biosciences, Piscataway, NJ, USA). PBMCs were labeled with 1.5 μM of carboxyfluorescein succinimidyl ester dye (CFSE, Molecular Probes, Eugene-OR) and cultured for five days in the presence of either 10 μg/mL of soluble *Leishmania* antigen (SLA),[9] 5 μg/mL of phytohaemagglutinin (PHA) or culture medium,.[10] Next, PBMCs were stained with CD4^+^ and CD8^+^ monoclonal antibodies conjugated with phycoerythrin (PE) and allophycocyanin (APC). Cell acquisition was performed using a FACSAria Flow-Cytometer (Becton Dickinson, CA, USA) and subsequently analyzed by Flowjo™ software (v7.6, Tree Star, Inc. 1997–2009). The cell division index (DI) was used to quantify the proliferation intensity of T-cell subsets (DI = 0.06 for CD4+ and 0.09 for CD8+ T-cells.) The frequencies of CD4^+^ and CD8^+^ T-cells producing intracellular cytokines were quantified using flow cytometry. PBMCs were cultured in the presence of SLA, PHA or culture medium for 18 h. Heat-inactivated human AB serum, brefeldin A and monensin were added to all cultures in the final four hours. Next, PBMCs were stained with anti-CD4-fluorescein isothiocyanate (FITC) and anti-CD8-APC, then permeabilized with PBS-BSA-Saponin 0.2% and incubated with anti-INF-γ-PE, anti-TNF-α-PE, and anti-IL-10-PE (Becton Dickinson, CA, USA). Plasma cytokine levels were quantified using the BD Cytometric Bead Array (CBA) Human Th1/Th2 Cytokine Kit II (San Jose, CA, USA).

## Case presentation

**Figure 1:**
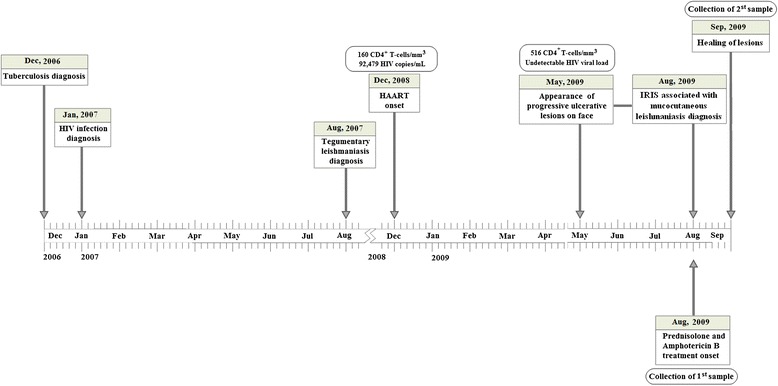
**A time line of clinical manifestation of a patient with mucocutaneous leishmaniasis as a manifestation of immune reconstitution inflammatory syndrome.**

**Figure 2:**
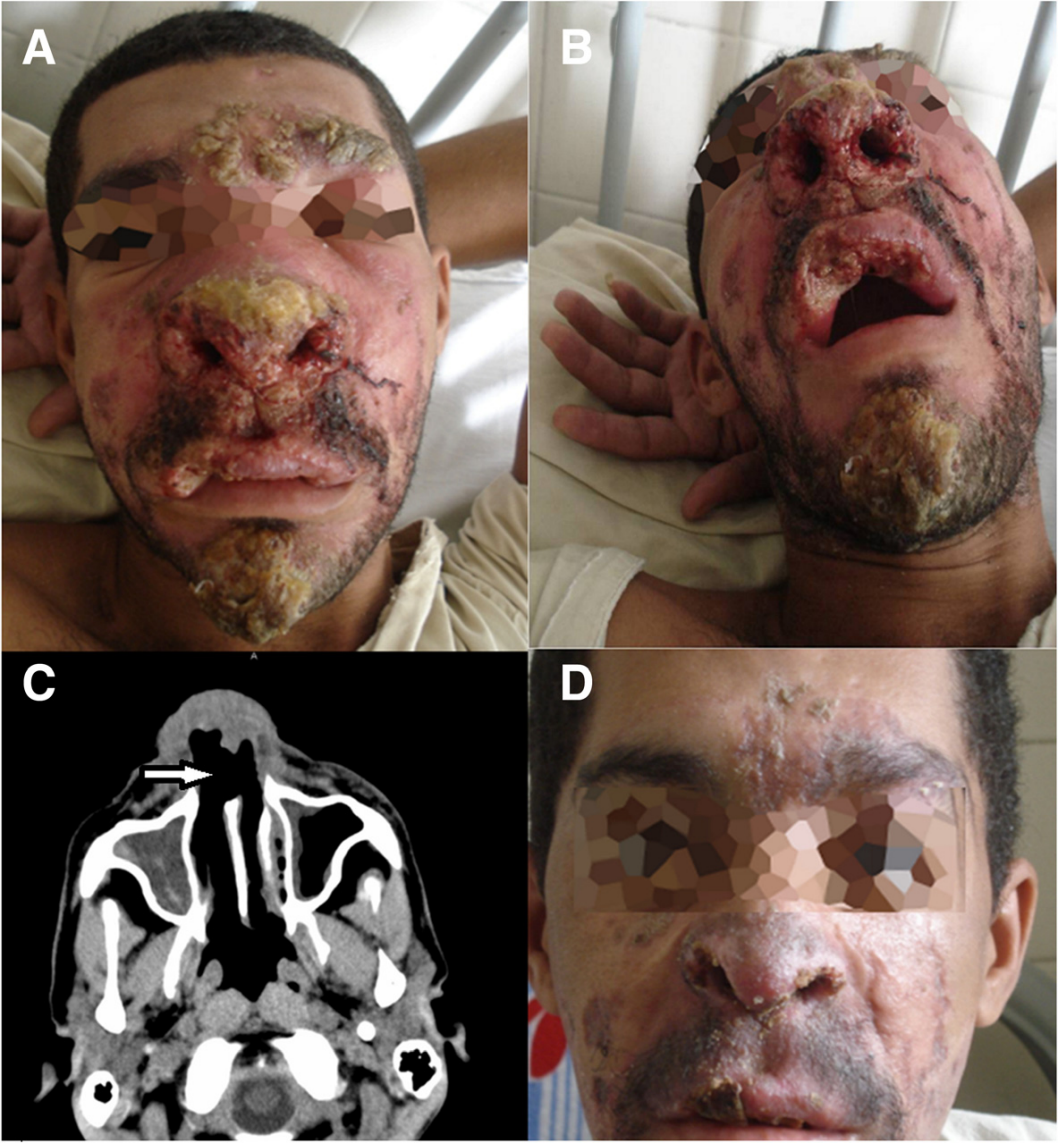
**An HIV-infected patient with mucocutaneous leishmaniasis as a manifestation of immune reconstitution inflammatory syndrome (IRIS).** In May 2009, five months after the initiation of HAART therapy, he presented with ulcerative lesions on his face in association with nasal obstruction. **A** and **B**: By August 2009, the lesions progressed and severe inflammation with pronounced swelling of the lips, nasal and mentum regions are pictured. **C**: Computerized tomography of the paranasal sinus cavities shows partial destruction of the nasal septum (white arrow) (August, 2009). **D**: Healing of lesions observed following treatment with Prednisolone and Amphotericin B (September 2009).

The patient, a 29-year-old HIV-1-infected male, reported being treated for pulmonary tuberculosis in 2006. In 2007, the patient’s serology for HIV tested positive. Eight months later, the patient reported an ulcerative lesion in his lower right limb, which was diagnosed as tegumentary leishmaniasis. He subsequently received pentavalent antimony therapy, resulting in the healing of this lesion. In December 2008, the patient began HAART therapy (zidovudine, lamivudine, and efavirenz). At this time, his CD4^+^ T-cell count was 160 cells/mm^3^ and viral load was 92,479 copies/mL. In May 2009, he presented with ulcerative lesions on his face in association with nasal obstruction. At this point, his CD4^+^ T-cell count was 516 cells/mm^3^ with an undetectable HIV viral load. The lesions subsequently progressed, resulting in severe inflammation characterized by a pronounced swelling of the lips, nasal and mentum regions. He was admitted to Hospital Professor Edgard Santos (HUPES) in August 2009 (Figure 1). The lesions were crusty in appearance and a partial destruction of the patient’s upper lip and nose was observed (Figure 2A and B). In addition, myiasis was observed in his necrotic lesions. Destruction of the nasal septum was confirmed by computerized tomography of the paranasal sinus cavities (Figure 2C).

A skin biopsy revealed the presence of granulomatous lesions. Caseous necrosis was identified by a cervical lymph node biopsy. Amastigote forms of *Leishmania spp.* were found in both skin and lymph node biopsies. The skin test for *Leishmania* was positive (15 mm) and indirect ELISA for soluble *L. brasiliensis* antigens was positive, while ELISA for rK39 was negative. IgG anti-*L. braziliensis* levels were measured using indirect ELISA for soluble *L. braziliensis* antigens. The specie determination was further confirmed using a serial real-time quantitative PCR assay system, as described by Weirather, JL, 2011 [11].

The patient was then diagnosed with mucocutaneous leishmaniasis as a manifestation of IRIS and HAART was discontinued. He was subsequently treated with 80 mg/day of Prednisolone and 1 mg/Kg/day of Amphotericin B from August to September 2009. After 15 days of treatment, improvement in the mucocutaneous lesions and in the patient’s cervical lymphadenopathy was observed (Figure 2D). After two months of hospitalization, the lesions had completely healed and the patient was discharged. HAART therapy was subsequently reintroduced.

### Immune response to *Leishmania* antigens during and after IRIS

**Figure 3:**
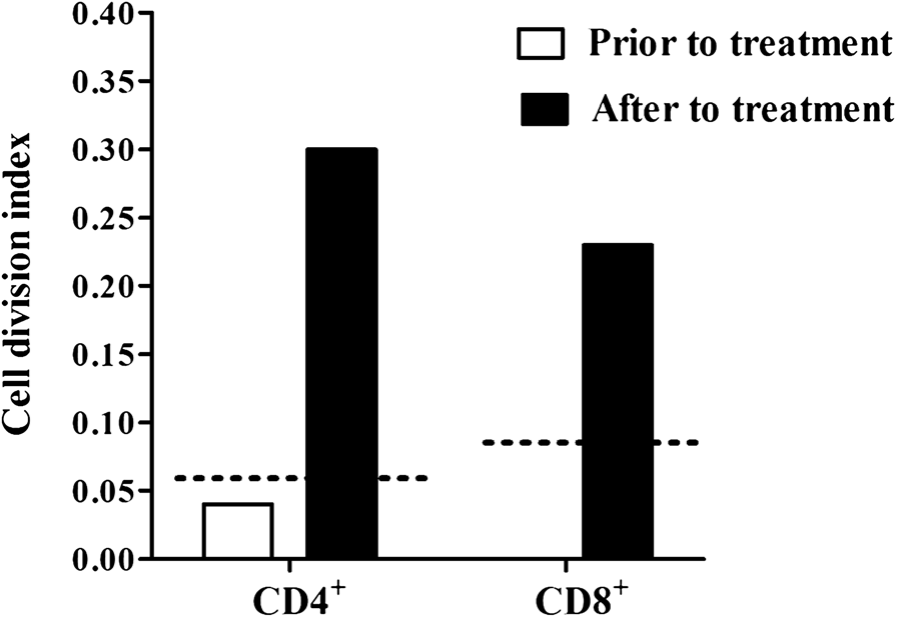
**Evaluation of CD4+ and CD8+ T-lymphocyte proliferation in response to soluble** ***Leishmania*** **antigens (SLA) in a patient with mucocutaneous leishmaniasis as manifestation of IRIS.** Blood samples were collected before (August 2009) and one day following the conclusion of Amphotericin B and corticosteroid treatment (September 2009). The cell division index was calculated using Flowjo™ software. White and black bars represent the T-cell proliferative responses to *Leishmania* antigens prior to and after Amphotericin B treatment, respectively. Dashes represent the threshold value with respect to a positive proliferative response (above 0.06 for CD4+ T-cell and above 0.09 CD8+ T-cell subset).

**Figure 4:**
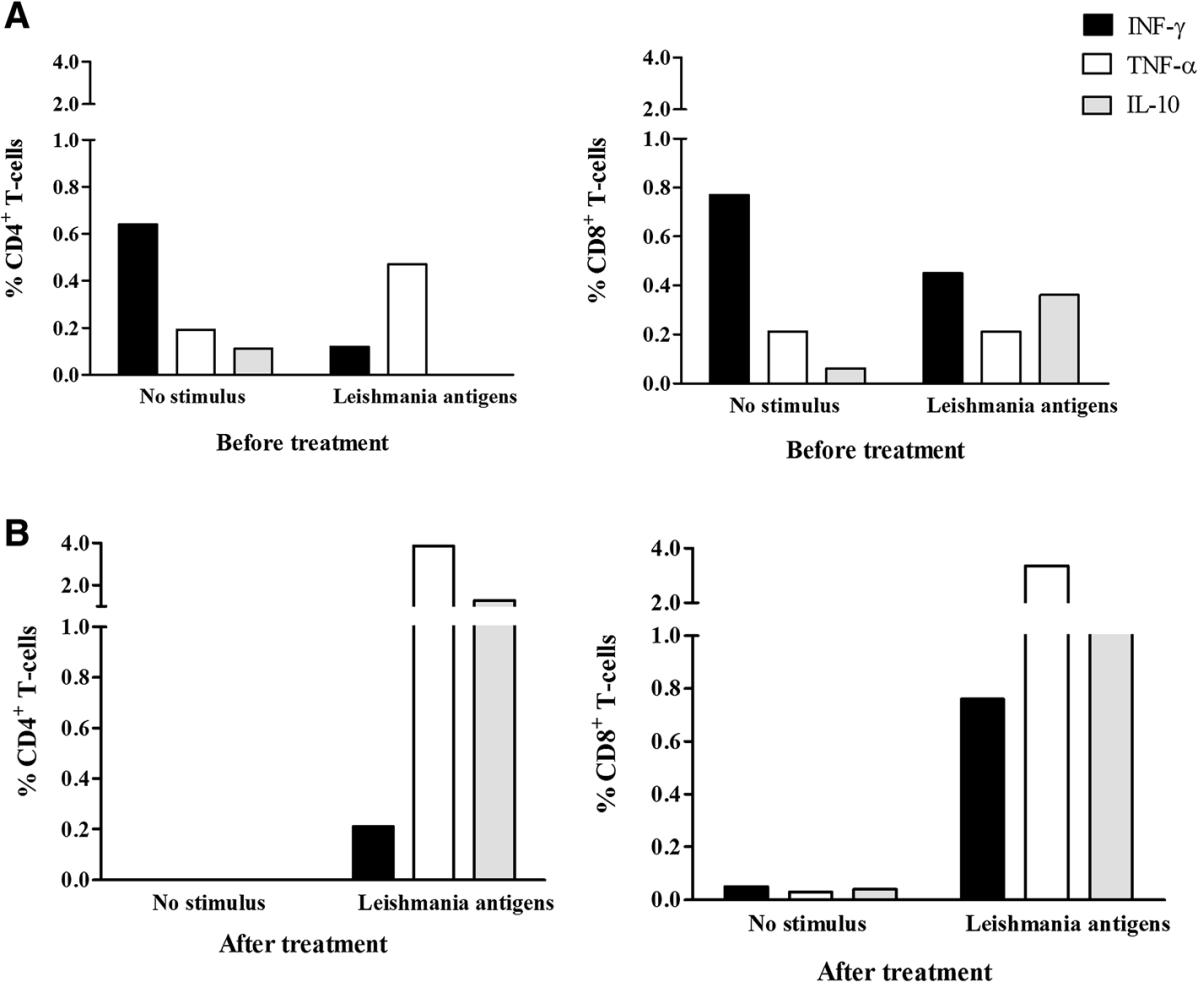
**Proportion of CD4+ and CD8+ T-cells producing intracellular IFN-γ** **, TNF-**α **and IL-10 after culturing without stimulus, and in the presence of SLA.** Blood samples were collected in August 2009 from a patient with mucocutaneous leishmaniasis as manifestation of IRIS before **(A)** and one day after the end **(B)** of Amphotericin B and corticosteroid treatment (September 2009).

Prior to treatment with Amphotericin B, the patient’s proliferative response to SLA was undetectable in both CD4^+^ and CD8^+^ T-cell subsets (Figure 3 – white bars). In the absence of SLA stimulus, the production of cytokines (IFN-γ, TNF-α and IL-10) by both CD4^+^ and CD8^+^ T-cells were similar under both conditions. In addition, IL-10 production was undetectable following SLA stimulation in the CD4^+^ T-cell subset (Figure 4A). Following leishmanial treatment, a proliferative response to SLA was observed in both CD4^+^ (DI: 0.3) and CD8^+^ T-cell subsets (DI: 0.2) (Figure 3 – black bars). In the absence of SLA stimulus, IFN-γ, TNF-α and IL-10 production by CD4^+^ T-cells was undetectable, and few CD8^+^ T-cells were observed producing these cytokines. By contrast, in response to SLA stimulation, a high proportion of CD4^+^ and CD8^+^ T-cells producing TNF-α and IL-10 was detected (Figure 4B). Production of IFN-γ was markedly higher in CD8^+^ cells in comparison with that of CD4^+^ T-cells. Plasmatic levels of INF-γ, TNF-α, IL-2 and IL-10 were higher at the conclusion of treatment with Amphotericin B, while IL-6 decreased, and the IFN-γ/IL-10 ratio rose from 0.0 to 3.6 over the course of treatment (Table 1).

**Table 1:** **Cytokine levels in the plasma of a patient with mucocutaneous leishmaniasis as manifestation of IRIS, before and after Amphotericin B treatment**

**Cytokine**	**Amphotericin B treatment**	
	**Before**	**After**
IFN-γ	0.0	47.7
TNF-α	11.1	17.8
IL-10	5.3	13.3
IL-2	15.0	22.5
IL-4	7.1	8.2
IL-6	17.6	10.6
IFN-γ/IL-10 ratio	0.0	3.6

Data are presented in pg/mL. Cytokine levels were quantified before and one day after the end of Amphotericin B and corticosteroid treatment.

## Discussion

Cutaneous leishmaniasis as a manifestation of IRIS may appearance, following the introduction of HAART and consequent restoration of immunity, as a new disease or as the progression of latent disease [12]. In the present report, the patient’s lesions developed five months after the initiation of antiretroviral therapy, concurrent with the recovery of the number of CD4^+^ T-cells.

To the best of our knowledge, only two other cases of patients infected with HIV and the clinical form of mucocutaneous leishmaniasis as a manifestation of IRIS have been described to date [6]. In both of these cases, disseminated skin lesions (on the arms, lower limbs and feet) and lesions in the nasal, oropharyngeal, as well as genital mucosa were reported. Although genital lesions have been reported in one-third of HIV/*Leishmania* co-infected patients [12,13], the patient described herein did not show genital involvement or widespread skin lesions. His lesions were restricted to the facial area, especially in the nasal and oropharyngeal mucosa, with intense inflammation resulting in destruction of the nasal septum.

Our results suggest that the mucosal damage resulting from mucocutaneous leishmaniasis as a manifestation of IRIS in this patient was correlated with an unspecific inflammatory milieu. During the course of IRIS, CD4^+^ and CD8^+^ T-lymphocytes produced very low levels of IFN-γ and TNF-α in response to *Leishmania* antigens, yet high levels of IFN-γ by both CD4^+^ and CD8^+^ T-cells were observed in the absence of antigen stimulation. Conversely, after the lesions healed, a specific immune response to *Leishmania* antigens was reestablished and a profound reduction in the spontaneous production of cytokines was observed. The decreased T-cell proliferation and low antigen-induced cytokine responses observed in this patient during the course of IRIS could be more suggestive of immunosuppression to *Leishmania* antigens than a hyper-responsive state. However, the fact that the lesions appeared at the same time the patient’s immune system demonstrated recovery (the CD4^+^ T lymphocyte count was higher than 500 cells/mm^3^ and HIV viral load was undetectable) is supportive of an IRIS diagnosis. Moreover, the initial clinical presentation of mucosal leishmaniasis, which appeared five months after HAART initiation, progressed to an intense inflammatory response with partial destruction of the nasal septum in less than four months.

During IRIS, the patient had a positive skin test for *Leishmania*, while *his antigen*-specific T-cell proliferation was undetectable. This discrepancy could be explained by the dynamic of immune restoration following HAART. The restoration of antigen-specific CD4^+^ T-cell responses *in vitro* is mostly correlated with CD4^+^ memory T-cell reconstitution; whereas the improvement of delayed type hypersensitivity is associated with the suppression of viraemia [14]. However, it was not possible to quantify antigen-specific central and effector memory CD4^*+*^ T-lymphocytes for this patient, during IRIS or after healing of lesions. In addition, an impairment of *in vitro* proliferative response to *Leishmania* antigens could be linked to the activation and exhaustion of immune system found during IRIS [15]. Yet, intrinsic differences among tests for measuring cellular immune function could explain these divergent results.

In the present case, the participation of a specific response to *Leishmania* antigens with respect to the development of skin and mucosal lesions cannot be excluded, since a *Leishmania*-specific response was not evaluated *in situ*. A specific recruitment of CD4^+^ and CD8^+^ T-cells to the ulcerative lesion is described in patients with both cutaneous and mucosal leishmaniasis [16,17]. Specifically, in the mucosal lesions it is observed high number of IFN-γ-producing cells [16]. Indeed, a decreased type-1 immune response to *Leishmania* antigens in peripheral blood is associated with intense recruitment of *Leishmania*-specific T-cells to the lesions, which is classically found in HIV-uninfected patients with disseminated leishmaniasis, yet cytokine production in tegumentary lesions was observed to be similar to that found in patients with mucocutaneous and cutaneous leishmaniasis [18]. Following healing, though CD4^+^ and CD8^+^ T-cells persist in treated lesions, the number of circulating antigen-specific CD4^+^ and CD8^+^ T-cells increases in peripheral blood [19,20]. Thus, in this patient the absence of proliferative response to *Leishmania* antigens during IRIS might be due to sequestration of T-cells inside lesions. After healing, these cells were redistributed to peripheral blood.

The elevated cytokine production observed in non-stimulated cells may be a consequence of activated memory CD4^+^ T-cells specific for pathogens other than *Leishmania.* These cells recirculate from lymphoid organs into the peripheral blood stream during the first two months of HAART [1,21]. Moreover, the chronic activation of the immune system by HIV itself may also contribute to the inflammatory state observed during the course of IRIS [22].

High levels of IL-6 and TNF-α have been previously described in patients with other infectious diseases in association with IRIS [23-25]. Additionally, elevated pro-inflammatory cytokine production is considered to be a predictor of IRIS development in HIV-infected patients at the onset of HAART [26]. Considering the plasma levels of TNF-α in two HIV-uninfected patients with active mucocutaneous leishmaniasis evaluated at our laboratory (22 pg/mL and 13 pg/mL) [27], the levels observed in this patient during IRIS were similar. Following treatment with Amphotericin B and corticosteroids, an increase in plasmatic IFN-γ, as well as in the IFN-γ/IL-10 ratio, was observed in the present case, while TNF-α remained stable. Although treatment with corticosteroids usually decreases the production of inflammatory cytokines [28], a decrease in the spontaneous production of IFN-γ and TNF-α was observed in this patient only *in vitro,* but not in his plasma. This finding may be due to the fact that post-treatment cytokine quantification was performed in the plasma by a single measurement just one day following the conclusion of treatment. It is possible that subsequent measurements would have found decreases in TNF-α in the plasma.

Furthermore, the nonspecific activation of the immune system could be a consequence of an impaired regulatory T-cell response or a decrease in the proportion of these cells, since IL-10 produced by *Leishmania*-specific CD4^+^ T-lymphocytes was not detected during the course of IRIS [8]. The proportion of regulatory T-cells (1.8%) in our patient prior to treatment was found to be low (data not shown). An imbalance between the effector and regulatory responses may also play a role in the pathogenesis of IRIS [29,30].

IRIS is characterized by an intense inflammatory reaction, leading to tissue destruction concurrent with an increase in the number of CD4^+^ T-cells following HAART. No convincing evidence has been presented in regard to whether CD4^+^ T-cells specific to opportunistic pathogens are deregulated during IRIS, much less if these cells are involved in the pathogenesis of IRIS. Moreover, the literature contains no reports that directly link CD4^+^ T-cells specific to *Leishmania* antigens with clinical manifestations of leishmaniasis in association with IRIS. However, several reports have shown a recovery of CD4^+^ T-cells specific to *Mycobacterium tuberculosis*, *Mycobacterium avium* complex and *Cryptococcal neoformans* in patients who developed IRIS in association with these infections [18,29,31]. Interestingly, another study found no clear association between the recovery of *M. tuberculosis*-specific CD4^+^ T-cells and tuberculosis in association with IRIS [29].

It has been suggested that the absence of T-cells, such as occurs in the course of AIDS, may lead to the growth of intracellular pathogens inside macrophages that never become fully activated, and thus exert no effector function. When antigen-specific CD4^+^ T-cells are restored following HAART, these cells may intensely stimulate macrophages and possibly other innate immune cells that produce large amounts of proinflammatory cytokines, resulting in inflammation and tissue destruction [31].

## Conclusion

In summary, the results presented herein suggest that *Leishmania*-associated IRIS occurred concurrently with the production of proinflammatory cytokines by unstimulated T-lymphocytes, which is supported by the absence of a specific host immune response against *Leishmania* antigens in the peripheral blood of this patient.

### Consent

Written informed consent was obtained from the patient for publication of this case report and any accompanying images. A copy of the written consent is available for review by the Editor of this journal.
